# Investigating Whether AI Will Replace Human Physicians and Understanding the Interplay of the Source of Consultation, Health-Related Stigma, and Explanations of Diagnoses on Patients’ Evaluations of Medical Consultations: Randomized Factorial Experiment

**DOI:** 10.2196/66760

**Published:** 2025-03-05

**Authors:** Weiqi Guo, Yang Chen

**Affiliations:** 1 School of Foreign Languages Renmin University of China Beijing China; 2 School of Journalism and Communication Renmin University of China Beijing China

**Keywords:** artificial intelligence, AI, medical artificial intelligence, medical AI, human–artificial intelligence interaction, human-AI interaction, medical consultation, health-related stigma, diagnosis explanation, health communication

## Abstract

**Background:**

The increasing use of artificial intelligence (AI) in medical diagnosis and consultation promises benefits such as greater accuracy and efficiency. However, there is little evidence to systematically test whether the ideal technological promises translate into an improved evaluation of the medical consultation from the patient’s perspective. This perspective is significant because AI as a technological solution does not necessarily improve patient confidence in diagnosis and adherence to treatment at the functional level, create meaningful interactions between the medical agent and the patient at the relational level, evoke positive emotions, or reduce the patient’s pessimism at the emotional level.

**Objective:**

This study aims to investigate, from a patient-centered perspective, whether AI or human-involved AI can replace the role of human physicians in diagnosis at the functional, relational, and emotional levels as well as how some health-related differences between human-AI and human-human interactions affect patients’ evaluations of the medical consultation.

**Methods:**

A 3 (consultation source: AI vs human-involved AI vs human) × 2 (health-related stigma: low vs high) × 2 (diagnosis explanation: without vs with explanation) factorial experiment was conducted with 249 participants. The main effects and interaction effects of the variables were examined on individuals’ functional, relational, and emotional evaluations of the medical consultation.

**Results:**

Functionally, people trusted the diagnosis of the human physician (mean 4.78-4.85, SD 0.06-0.07) more than medical AI (mean 4.34-4.55, SD 0.06-0.07) or human-involved AI (mean 4.39-4.56, SD 0.06-0.07; *P*<.001), but at the relational and emotional levels, there was no significant difference between human-AI and human-human interactions (*P*>.05). Health-related stigma had no significant effect on how people evaluated the medical consultation or contributed to preferring AI-powered systems over humans (*P*>.05); however, providing explanations of the diagnosis significantly improved the functional (*P*<.001), relational (*P*<.05), and emotional (*P*<.05) evaluations of the consultation for all 3 medical agents.

**Conclusions:**

The findings imply that at the current stage of AI development, people trust human expertise more than accurate AI, especially for decisions traditionally made by humans, such as medical diagnosis, supporting the algorithm aversion theory. Surprisingly, even for highly stigmatized diseases such as AIDS, where we assume anonymity and privacy are preferred in medical consultations, the dehumanization of AI does not contribute significantly to the preference for AI-powered medical agents over humans, suggesting that instrumental needs of diagnosis override patient privacy concerns. Furthermore, explaining the diagnosis effectively improves treatment adherence, strengthens the physician-patient relationship, and fosters positive emotions during the consultation. This provides insights for the design of AI medical agents, which have long been criticized for lacking transparency while making highly consequential decisions. This study concludes by outlining theoretical contributions to research on health communication and human-AI interaction and discusses the implications for the design and application of medical AI.

## Introduction

### Background

Artificial intelligence (AI) is considered a revolutionary technology for the health care sector. Analysts have forecasted massive growth in the global AI health care market over the next decade [1]. AI is now used in medical scenarios in 2 forms: either to make independent medical decisions (ie, autonomous AI) or to collaborate with human physicians and make decisions with human involvement (ie, human-involved AI) [2]. While ideally, AI diffusion promises to improve diagnostic accuracy, save human effort, and increase patient convenience, empirical research is needed to assess the merits and limitations of medical AI [3], especially from a patient-centered perspective [4]. This perspective is significant because AI as a technological solution does not necessarily improve the patient’s confidence in the diagnosis and adherence to treatment at the functional level, establish meaningful interactions between the medical agent and the patient at the relational level, and stimulate positive emotions in the patient and reduce pessimism at the emotional level. This leads to the guiding research question (RQ) of this study as follows: From the patient’s perspective, can autonomous AI or human-involved AI replace the role of human physicians at the functional, relational, and emotional levels?

Previous studies and practical necessities motivate this study in several ways. First, although previous studies have explored human-AI interactions guided by some established frameworks, such as the computers are social actors (CASA) paradigm or algorithm aversion theory, by analyzing scenarios of web-based content production and moderation, consumer behavior, and so on [5,6], there is still a lack of empirical evidence as to whether these conclusions can adequately address the complexities of the medical scenario. Little research has systematically examined, from a user-centered approach, the functional, relational, and emotional evaluations of human-AI interactions in medical scenarios, which may yield different conclusions from other scenarios when contextual factors, such as the severe consequences of medical interventions and the attributes of the disease, are taken into account. In addition, few studies have tested user evaluations of human-involved AI, especially vis-à-vis AI and humans; however, this model of human-AI collaboration is increasingly prevalent in medical scenarios.

Second, a crucial distinction between human-AI interaction and human-human interaction in the health domain is that of the human touch, a quality traditionally associated with human physicians, which may affect the patient’s evaluation of the medical consultation. Therefore, we incorporated health-related stigma into the analysis of human-AI interaction. Stigma is often associated with human-human interactions, and in the medical scenario, it may lead to patients not seeking a diagnosis or withholding information from physicians. However, this also presents opportunities to introduce AI in a manner that eliminates the human element from medical services. Earlier studies have primarily examined health-related stigma and human-AI interaction separately [7,8]; however, by linking these two variables, this study is able to address the broader question of how disease attributes and communicator characteristics influence the preference for machines over humans in medical scenarios and whether dehumanization is sometimes beneficial in health care.

Third, previous theories have consistently emphasized the accuracy and convenience of AI-powered systems as reasons for preferring AI over humans [9]. In the medical scenario, however, is it enough for AI to merely be accurate and efficient to replace human practitioners? In particular, given the long-standing issue of accurate diagnoses being overlooked due to a lack of explanation and low patient comprehension in traditional patient-physician communication [10] and the black-box nature of AI that systematically ignores explanations for users [11], it is imperative to investigate whether providing explanations of the diagnostic decision-making process enhances effective communication between the medical agent and the patient. This could shed light on how AI-powered medical agents should be designed from a user-centered approach.

To answer these questions, an experiment was conducted based on the theories of human-computer interaction (HCI), human-machine communication (HMC), and health communication. We constructed different scenarios where the source of medical consultation (ie, AI vs human-involved AI vs human), the stigma of the health issue (ie, high vs low), and the explanation of diagnosis (without vs with explanation) were manipulated and examined their individual and interaction effects on people’s functional, relational, and emotional evaluations of the medical consultation. On the basis of the findings, we outlined the theoretical contributions to the understanding of human-AI interaction and health communication and practical implications for the principles of human-AI collaboration in health care and the design of AI-powered medical agents.

### Literature Review

#### AI, Human-Involved AI, and Humans as Sources of Medical Consultation

AI is considered promising for enhancing the efficiency, accuracy, and quality of medical services. People are increasingly turning to AI-powered instruments to understand their symptoms, seek diagnoses, and explore treatment options, which previously occurred between patients and human physicians in offline clinical environments [12]. Currently, there are 2 paradigms for the application of AI in medical consultation and diagnosis. Patients may encounter autonomous AI that replaces human physicians and makes medical decisions independently [2]. They can also opt for human-involved AI, where human physicians collaborate with AI, participate in the medical decision-making loop, and monitor AI’s diagnostic results and treatment recommendations [13,14]. Human-involved AI is an intermediate paradigm between autonomous AI and human practitioners, synthesizing the functional and relational characteristics of both. Despite the growing importance of AI, few studies have systematically examined people’s functional, relational, and emotional evaluations of AI and human-involved AI versus humans in medical settings, along with the merits and shortcomings of the 3 medical agents in disease diagnosis, which are the focus of this study.

Existing HCI and HMC theories offer conflicting predictions regarding how users perceive AI versus humans. The CASA paradigm suggests that people apply the same social rules and expectations to machines as to humans despite knowing that they are inanimate agents. The implication is that there should be no difference in users’ perceptions of AI and humans [15]. However, as human-AI interactions become more common and technological affordances continue to advance, some recent evidence does not support the CASA paradigm [16]. Another framework based on the concept of machine heuristic suggests that people should favor AI as a source of communication over humans. The argument is that when people interact with machines, they tend to use mental shortcuts and apply general stereotypes of machines, such as being objective, accurate, unbiased, and predictive, thus viewing AI and its decisions as more favorable compared to human decisions [17]. Evidence of such AI preferences has been reported in health, media, and organizational settings, among others, in both low-risk and high-risk contexts [18-21]. In contrast, the algorithm aversion literature argues that people are suspicious of AI decision makers for several reasons, including the lack of transparency of algorithms in the decision-making process and the absence of human expertise in certain domains [22]. Accordingly, people prefer human decision makers to AI, especially for decisions that are traditionally made by humans, despite acknowledging the fact that machines’ overall performance is superior to that of humans [13].

Whether these theories could capture the complexities of the medical scenario remains unclear, as many studies approach this question by analyzing scenarios of web-based content moderation, media production, or consumer behaviors. In addition, little evidence exists to test user perceptions of human-involved AI, especially vis-à-vis AI and humans; however, this model of human-AI cooperation is very common in medical scenarios. Inspired by Guzman and Lewis [23], this study regards medical AI and human-involved AI as communicators similar to human physicians, rather than simple technologies mediating human interactions and explores users’ functional, relational, and emotional evaluations of the 3 agents in a medical setting.

Regarding users’ functional evaluations of the agents, the machine heuristic argument posits that medical AI exhibits a superior level of information processing ability compared to humans. AI trains and refines its decision-making models based on vast quantities of medical data and adheres to a consistent decision-making process that yields rational solutions, which could culminate in higher functional evaluations of AI from users [9]. Conversely, the algorithmic aversion literature suggests that because disease diagnosis and treatment are traditionally performed by humans, users may perceive human physicians as more authoritative and reliable than medical AI [24]. When AI and human physicians collaborate in diagnosis (ie, human-involved AI), it is expected that the benefits of both approaches will be integrated to provide users with fast, accurate, and authoritative medical decisions and thus generate the highest functional evaluations.

Regarding relational evaluations, machine heuristic may make users less likely to consider relational attributes of AI during interactions, making it more difficult for medical AI to build relationships with users in the way that human physicians do. However, the CASA paradigm offers opposing predictions in this regard [25]. When the communicator is a human-involved AI, the medical consultation involves both human attributes of interpersonal communication and elements of dehumanized HMC. Therefore, the user’s relational evaluation of the agent is hypothesized to be intermediate between that of an autonomous AI and a human physician. In addition to functional and relational evaluations, it is essential to understand how different medical agents induce emotional responses in patients. Are different types of medical agents equally effective in helping people comprehend their health conditions (ie, functionally helpful), providing empathetic support (ie, relationally supportive), and thus eliciting positive emotional responses (eg, optimism) and reducing negative emotions (eg, guilt about their conditions)?

Taken together, we proposed RQ1: Are there differences in people’s functional, relational, and emotional evaluations of medical consultations with different medical agents (AI vs human-involved AI vs human)?

#### Health-Related Stigma and Its Influence on Medical Consultations

Stigma is a mark or condition that is related to social dishonor, stereotyping, and discriminatory beliefs [26,27]. One health issue that is typically stigmatized is HIV infection or AIDS. Patients with stigmatized diseases, such as AIDS, are reportedly hesitant to seek treatment due to the social shame attached to these diseases and the unavoidable disclosure of private information, such as risky sexual behaviors, during medical consultations [28]. Face-to-face consultations can be particularly uncomfortable, causing patients to withhold relevant information or lie to physicians [29]. Consequently, as digital health care applications proliferate, patients with stigmatized health conditions may turn to AI-powered medical agents rather than human physicians for medical services [12]. This means that health-related stigma may influence health care preferences, thereby altering the functional, relational, and emotional evaluations of consultations with different medical agents.

The technological affordances of AI facilitate new experiences in medical consultations for people with stigmatized diseases, compared to interactions with human physicians. Medical AI promises greater anonymity and privacy. If patients want to consult without revealing their identity, AI has an advantage over clinical visits or phone consultations [30]. Furthermore, people tend to view AI as a nonjudgmental tool and thus feel more comfortable disclosing personal health information, such as high-risk sexual conduct, to a medical AI than to human physicians [27,31]. This indicates the potential necessity for dehumanization in medical consultations, particularly when stigma is present [29].

This study examined the effect of stigma on preferences for medical agents in the context of 2 conditions, AIDS and heart disease, the former being more stigmatized and the latter less. Previous research has demonstrated the influence of disease severity on patient preferences for medical agents [32]. This study aimed to control for the effect of disease severity and focus on the role of stigma. While AIDS and heart disease are similar in terms of disease severity, as both have serious health consequences and require accurate treatment, they diverge in terms of stigma. AIDS is typically stigmatized, whereas heart disease is not subject to cultural judgments about one’s private life and is not infective. On the basis of the aforementioned theoretical discussions, we expected that in stigma-laden conditions (eg, AIDS), where patients need instrumental guidance and prefer that the medical agent refrains from judging their lifestyles, reducing the human element in medical consultations would enhance patients’ evaluations of the medical experience. When stigma was not involved (eg, heart disease), we expected that patients would still prefer human expertise and human touch during medical consultations and would give higher functional, relational, and emotional ratings to human physicians than to autonomous AI or human-involved AI.

RQ2 is as follows: How does health-related stigma (AIDS vs heart disease) affect the functional, relational, and emotional evaluations of the medical consultation?

#### Explanations of Diagnoses and Evaluation of Medical Consultation

The effectiveness of physician-patient communication is critical to patients’ evaluations of medical consultations and the achievement of positive health outcomes. The physician’s ability to explain the condition and diagnosis to the patient is of particular significance [33]. Explanation is defined as the details or reasons given to facilitate comprehension. For health care professionals, effective diagnostic explanations require interpreting symptoms and justifying diagnostic and treatment decisions. Operationally, the diagnostic explanation in this study encompassed 4 aspects. First, patients should be informed of the symptom descriptions required to confirm their conditions. Second, the rationale for the diagnosis formulated must be explained, which includes clarifying the range of possible diagnoses based on the symptom descriptions and the reasons why a specific diagnosis is made. Third, different treatment options should be provided and explained, and a treatment recommendation should be offered. Finally, the efficacy of the relevant medication and the suggested medication should be explained.

In traditional clinical visits, patients have complained about the lack of explanation of diagnosis and treatment [10]. Sometimes, patients must turn to web-based communities to decipher the medical knowledge and reasoning embedded in medical disclosure documents [34]. Following this line of reasoning, as AI medical agents become more prevalent, the following questions arise. Do people have the same expectations when using medical AI and human-involved AI? If providing an explanation of the diagnosis enhances people’s evaluations of medical consultations, does this approach to enhancing physician-patient communication differ between different types of medical agents (ie, medical AI, human-involved AI, and human physician) and between different types of diseases (ie, highly stigmatized and less stigmatized diseases)? This study aimed to answer these questions.

Owing to the black-box nature of AI, decisions made by AI-involved systems are often criticized for lacking transparency and accountability [11]. When making high-impact decisions such as medical diagnosis, human understanding of the reasoning behind the decision-making process without the need to comprehend the internal structure or underlying algorithms, which is defined as the principle of intelligibility of explainable AI, should be helpful and satisfying to content recipients [35], thereby enhancing the functional, relational, and emotional evaluations of the human-AI interaction. For medical AI to replace human practitioners, being accurate is not enough. Empirical evidence indicates that an accurate diagnosis, without explanation or justification, may be ignored by patients, even if made by a highly competent physician, let alone by AI systems, which have long been criticized as unsafe due to their lack of explicability [4,36,37]. Therefore, we hypothesized that providing explanations of the diagnosis, whether by a human physician, medical AI, or human-involved AI, would improve patients’ functional, relational, and emotional evaluations of the medical consultation. Furthermore, we predicted that the greater the involvement of AI in the diagnosis, the more patients would require diagnostic explanations due to the increased black-box problem associated with AI. This, in turn, may affect their evaluations of the medical consultation on all 3 dimensions.

Regarding the interaction effect of health-related stigma and diagnosis explanation on patients’ evaluations of medical consultations, as previously discussed, regardless of the type of the medical agent, when diagnosis explanations are provided, patients with stigmatized and nonstigmatized conditions will similarly have a better understanding of their conditions and higher ratings of medical consultations [33]. If the medical agent does not explain the diagnosis, patients with a nonstigmatized condition may be less satisfied with the diagnosis, especially if the consultation is with an AI-involved system rather than a human physician. In contrast, people with stigmatized diseases are more likely to avoid further discussion with the medical agent, which potentially results in the revelation of more personal and stigmatized health information. Nevertheless, they are compelled to seek instrumental medical services due to the high risk of their health conditions [29]. Therefore, even if the medical agent only offers a diagnosis and treatment options but does not provide any explanation of the decision-making process, patients with stigmatized health issues are more likely to accept the diagnosis without requiring transparency.

Taken together, we proposed RQ3: Does providing explanations of the diagnosis improve the functional, relational, and emotional evaluations of the medical consultation? Do the 3 types of medical agents (ie, medical AI, human-involved AI, and human physician) and the 2 types of diseases (ie, highly stigmatized and less stigmatized diseases) differ in this path to improving the effectiveness of physician-patient communication?

## Methods

### Research Design

A 3 (consultation source: AI vs human-involved AI vs human) × 2 (health-related stigma: high vs low) × 2 (diagnosis explanation: without explanation vs with explanation) factorial experiment was conducted in November 2023 to test the effects of medical consultation source, health-related stigma, and diagnosis explanation on the functional, relational and emotional evaluations of human-AI interaction in a diagnostic setting. Consultation sources and health-related stigma are between-subject factors. Diagnosis explanation is a within-subject factor. Data were analyzed using SPSS Statistics (IBM Corp), consisting of descriptive statistics, the 2-tailed *t* test, and ANOVA.

### Ethical Considerations

Institutional review board approval was obtained from Renmin University of China. Undergraduate students participated in the study for partial course credits. Before collecting data, we asked for participants’ consent, explained the experiment’s purpose and the use of data, and assured them that participation was completely voluntary and anonymous. The data were anonymized, containing no identifiable information about the participants.

### Procedure and Stimuli

**Figure 1:**
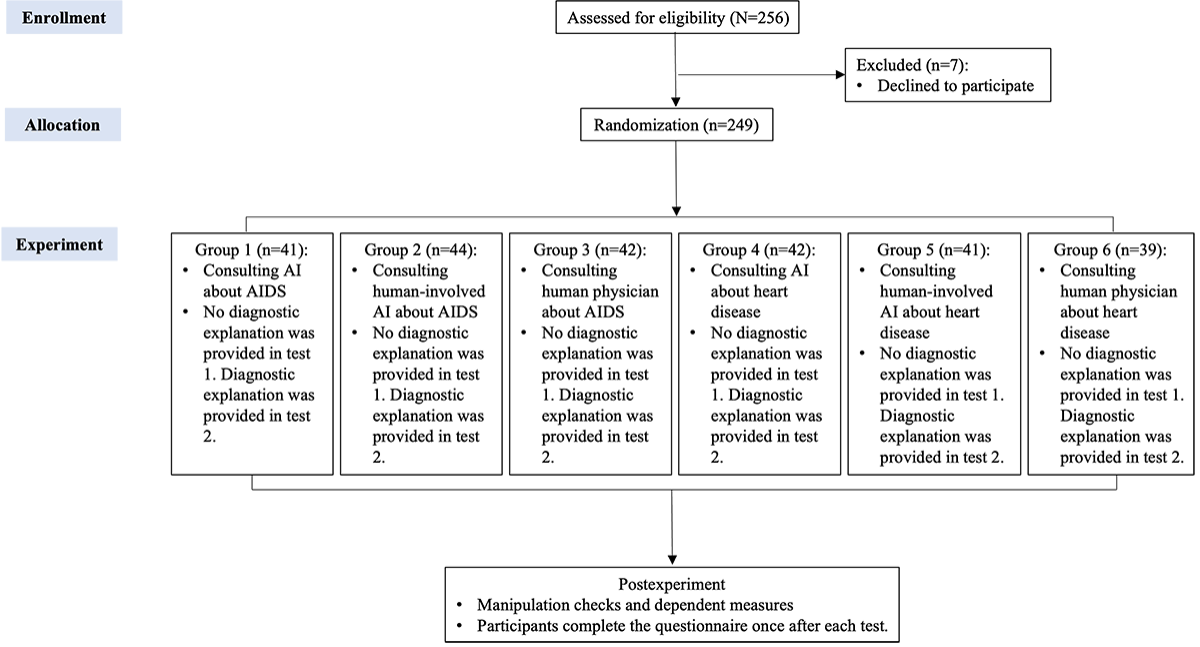
Experiment flow. AI: artificial intelligence.

Figure 1 illustrates the flow of the experiment. A total of 249 participants (mean age 18.67, SD 0.972 y; n=65, 26.1% male) were randomly assigned to 1 of the 6 experimental groups, which were conducted offline in 6 laboratories. Each participant was presented with condition-specific stimulus stories and given adequate reading time. Stimulus stories are conversations between an information seeker (concerned about AIDS or heart disease) and 1 of the 3 medical agents (an autonomous medical AI, a human-involved medical AI, or a human physician), presented in text form and typeset in a common one-to-one web-based chat interface (refer to Multimedia Appendix 1 for an example). Participants were first required to read an introduction in which they were informed that they had recently been unwell and wished to confirm their condition and obtain advice on treatment through a web-based medical consultation.

Then, to manipulate the consultation source, participants read prompts introducing the medical agent, such as “You are having a consultation with a medical AI. It learns from past medical data of patients with the same condition, makes a diagnosis based on the symptoms you’ve described, and offers treatment options. Human physicians are not involved in diagnosis, medication recommendations or monitoring of the consultation process” (the autonomous AI scenario). We also emphasized the manipulation of the source in the conversation scripts by referring to the medical agent as autonomous AI, human-involved AI, or human physician.

We manipulated health-related stigma through the conversation between the medical agent and the information seeker, with the high-stigma scenario involving symptoms of AIDS and the low-stigma scenario involving symptoms of heart disease. In a question-and-answer format, the patient described the symptoms and provided responses to the queries posed by the medical agent about his or her medical history. The medical agent proceeded to offer a diagnosis of the condition and treatment options. Participants consulting for the same condition received the same diagnosis and treatment options, either from an autonomous AI, a human-involved AI, or a human physician. We adapted the conversations based on the output of several leading medical AI models regarding AIDS and heart disease, also incorporating authentic records of web-based consultations provided by human physicians concerning the 2 diseases. The materials for the AIDS and heart disease groups differed in the patients’ symptom descriptions, medical history, and the medical agent’s diagnosis and treatment. The structure and content of the rest of the conversation were the same.

Each participant read 2 stimulus stories. The first reading was a brief conversation in which the medical agent made a diagnosis and offered treatment options but did not explain the rationale for the diagnosis. The second reading was an extended conversation in which the medical agent explained the diagnostic decision-making process in more detail, including the analysis of the symptoms, the clarification of potential diagnoses, the reasons why a specific diagnosis was made, and the analysis of treatment options and relevant medications. At the end of each reading, participants completed a questionnaire including manipulation checks and dependent variables.

### Dependent Measures

Functional measures of the medical consultation address how participants perceive the medical agent within its intended role [23]. Informed by the findings of Lew and Walther [38] and Appelman and Sundar [39], we explored 3 functional aspects of communication between the medical agent and the patient. *Source credibility* (mean 4.66, SD 0.88; Cronbach α=0.73) was measured with 4 items. Participants rated whether the source was trustworthy, competent, knew a lot, and had goodwill [38,40]. *Message credibility* (mean 4.61, SD 0.75; Cronbach α=0.81) was assessed using the 3-item message credibility scale [39], examining whether the messages were accurate, authentic, and believable. *Persuasive effect* (mean 4.45, SD 1.16; Cronbach α=0.83) was measured with 5 items [41], examining the extent to which participants would accept the diagnosis, follow the treatment advice, take the medication, seek more information from the medical agent in the future, and schedule another appointment with the agent if necessary. All items were rated on a 7-point scale (1=strongly disagree and 7=strongly agree).

Relational measures of the diagnostic experience pertain to how participants perceive the medical agent in relation to themselves [23]. Consistent with the findings of Croes and Antheunis [42] and Ahn et al [43], this study examined 3 relational aspects of medical agent–patient communication. *Perceived empathy* (mean 4.07, SD 1.34; Cronbach α=0.81) was measured with 5 statements (eg, “The medical agent came across as empathic”; 1=strongly disagree and 7=strongly agree) [42]. *Self-disclosure* (mean 4.35, SD 1.78; Cronbach α=0.87) was assessed using 4 statements [44], such as “I feel comfortable disclosing personal information when interacting with the medical agent” (1=strongly disagree and 7=strongly agree). *Psychological distance* (mean 2.99, SD 1.59) was measured with the single item of Inclusion of Other in the Self Scale [45]. Participants chose the picture that best represented their relationship with the medical agent (1=farthest and 7=closest).

Participants’ emotional responses to the medical consultation were examined by asking, “Do the following statements describe your feelings after the conversation with the medical agent?” We focused on 2 emotional responses that had opposing valences and were most relevant in the context of medical consultations: feeling optimistic and feeling guilty. Each response was measured with a single item rated on 7 points (1=no such emotion and 7=the emotion is extremely strong).

Participants reported demographic information at the end of the experiment. Sex was entered as a covariate in data analysis.

## Results

### Manipulation Check

As a manipulation check, participants answered three questions about their agreement with the statements after exposure to the assigned stimulus: (1) the medical agent is an autonomous AI, (2) the disease is socially stigmatized, and (3) the diagnostic explanation is provided. Autonomous AI (mean 2.71, SD 1.33) was rated as more autonomous than human-involved AI (mean 3.20, SD 1.06) and the human physician (mean 5.52, SD 1.75; *F*_2,240_=91.37; *P*<.001). AIDS (mean 5.17, SD 0.79) was rated as more stigmatized than heart disease (mean 2.68, SD 1.08; t_220_=20.69; *P*<.001). The second scenario (with diagnosis explanation; mean 6.92, SD 0.70) was rated as having more explanation than the first scenario (no explanation; mean 1.65, SD 1.87; t_219_=–37.99; *P*<.001). The results indicated effective manipulation of consultation sources, health-related stigma, and diagnosis explanation.

### Testing the Influence of the Source of Medical Consultation

RQ1 asked about the influence of consultation sources on the functional, relational, and emotional evaluations of the medical consultation. Regarding the functional evaluations, the main effect of consultation source emerged for source credibility (*F*_2,240_=7.75; *P*=.001; partial η^2^=0.06), message credibility (*F*_2,240_=9.52; *P*<.001; partial η^2^=0.07), and persuasive effect (*F*_2,240_=10.78; *P*<.001; partial η^2^=0.08). Post hoc tests (Bonferroni) indicated that diagnoses made by AI and by human-involved AI had significantly lower source credibility, message credibility, and persuasive effect as compared to those made by humans, but no significant differences were noted between the ratings of AI and human-involved AI (Table 1). Concerning the relational ratings, there were no differences in perceived empathy (*F*_2,240_=1.75; *P*=.18), self-disclosure (*F*_2,240_=0.63; *P*=.54), or psychological distance (*F*_2,240_=2.65; *P*=.07) between the 3 medical agents. The main effect of the consultation source did not exist for the 2 emotional responses: feeling optimistic (*F*_2,240_=0.34; *P*=.71) and feeling guilty (*F*_2,240_=1.04; *P*=.35).

**Table 1:** The main effect of consultation sources on the functional, relational, and emotional evaluations of the medical consultation.

			AI^a^, mean (SD)		Human-involved AI, mean (SD)		Human, mean (SD)		*F* test (*df*)		*P* value		Bonferroni test^b^
**Functional evaluations**													
	Source credibility	4.55 (0.06)		4.56 (0.06)		4.85 (0.06)		7.75 (2, 240)		.001		1<3, 2<3, and 1<2^c^	
	Message credibility	4.47 (0.07)		4.52 (0.07)		4.85 (0.07)		9.52 (2, 240)		<.001		1<3, 2<3, and 1<2^d^	
	Persuasive effect	4.34 (0.07)		4.39 (0.07)		4.78 (0.07)		10.78 (2, 240)		<.001		1<3, 2<3, and 1<2^e^	
**Relational evaluations**													
	Perceived empathy	4.06 (0.08)		3.97 (0.08)		4.17 (0.08)		1.75 (2, 240)		.18		NS^f^	
	Self-disclosure	4.28 (0.11)		4.30 (0.11)		4.44 (0.11)		0.63 (2, 240)		.54		NS	
	Psychological distance	2.82 (0.12)		2.94 (0.12)		3.20 (0.12)		2.65 (2, 240)		.07		NS	
**Emotional responses**													
	Optimistic	3.61 (0.1)		3.64 (0.1)		3.72 (0.1)		0.34 (2, 240)		.71		NS	
	Guilty	3.21 (0.1)		3.06 (0.1)		3.26 (0.1)		1.04 (2, 240)		.35		NS	

^a^AI: artificial intelligence.

^b^1 indicates mean value of AI group, 2 indicates mean value of human-involved AI group, and 3 indicates mean value of human group.

^c^1<3 (*P*=.002), 2<3 (*P*=.003), and 1<2 (*P*=.99).

^d^1<3 (*P*<.001), 2<3 (*P*=.002), and 1<2 (*P*=.99).

^e^1<3 (*P*<.001), 2<3 (*P*=.001), and 1<2 (*P*=.99).

^f^NS: not significant.

### Testing the Influence of Health-Related Stigma

RQ2 explored whether the stigma of the health issue (ie, AIDS vs heart disease) would directly influence or interact with the effect of consultation source on participants’ evaluations of the diagnosis. Results demonstrated that there were no significant differences in source credibility (*F*_1,240_=3.22; *P*=.07), message credibility (*F*_1,240_=0.01; *P*=.94), persuasive effect (*F*_1,240_=1.46; *P*=.23), perceived empathy (*F*_1,240_=0.27; *P*=.6), self-disclosure (*F*_1,240_=2.87; *P*=.09), or psychological distance (*F*_1,240_=0.02; *P*=.89) between the 2 diseases, indicating that the functional and relational evaluations were not significantly influenced by health-related stigma. Regarding the emotional responses, the main effect of health-related stigma emerged for feeling guilty (*F*_1,240_=44.55; *P*<.001; partial η^2^=0.16) but not for feeling optimistic (*F*_1,240_=1.42; *P*=.24). Post hoc tests (Bonferroni) revealed that the level of guilt was lower in the heart disease scenario (mean 2.79, SD 0.08) than in the AIDS scenario (mean 3.57, SD 0.08). The interaction effect of consultation source and health-related stigma did not appear for any of the dependent variables.

### Testing the Influence of Diagnosis Explanation

RQ3 investigated whether providing an explanation of the diagnosis would enhance the evaluation of the medical consultation and whether there were differences among the 3 medical agents and among the 2 types of diseases. First, the main effect of diagnosis explanation emerged for source credibility (*F*_1,240_=21.48; *P*<.001; partial η^2^=0.08), message credibility (*F*_1,240_=16.35; *P*<.001; partial η^2^=0.06), persuasive effect (*F*_1,240_=16.29; *P*<.001; partial η^2^=0.06), perceived empathy (*F*_1,240_=20.13; *P*<.001; partial η^2^=0.08), self-disclosure (*F*_1,240_=4.01; *P*=.04; partial η^2^=0.02), and psychological distance (*F*_1,240_=16.68; *P*<.001; partial η^2^=0.07). Post hoc tests (Bonferroni) revealed that providing an explanation of the diagnosis significantly increased all the functional and relational evaluations of the medical consultation (Table 2). Regarding emotional responses, the main effect of diagnosis explanation emerged for feeling optimistic (*F*_1,240_=15.27; *P*<.001; partial η^2^=0.06) and feeling guilty (*F*_1,240_=4.87; *P*=.03; partial η^2^=0.02). Participants had significantly higher levels of optimism and lower levels of guilt when the diagnosis was explained (Table 2).

**Table 2:** The main effect of diagnosis explanation on the functional, relational, and emotional evaluations of the medical consultation.

		No explanation, mean (SD)	With explanation, mean (SD)	*F* test (*df*)	*P* value	Bonferroni test^a^
**Functional evaluations**						
	Source credibility	4.2 (0.05)	5.12 (0.04)	21.48 (1, 240)	<.001	1<2
	Message credibility	4.26 (0.05)	4.97 (0.04)	16.35 (1, 240)	<.001	1<2
	Persuasive effect	4.07 (0.05)	4.94 (0.05)	16.29 (1, 240)	<.001	1<2
**Relational evaluations**						
	Perceived empathy	3.36 (0.06)	4.77 (0.05)	20.13 (1, 240)	<.001	1<2
	Self-disclosure	4.15 (0.07)	4.53 (0.07)	4.01 (1, 240)	.04	1<2
	Psychological distance	2.3 (0.07)	3.67 (0.09)	16.68 (1, 240)	<.001	1<2
**Emotional responses**						
	Optimistic	3.07 (0.06)	4.24 (0.07)	15.27 (1, 240)	<.001	1<2
	Guilty	3.37 (0.07)	2.99 (0.06)	4.87 (1, 240)	.03	2<1

^a^1 indicates the mean value of no diagnostic explanation and 2 indicates the mean value of providing a diagnostic explanation.

Furthermore, a significant interaction effect appeared between consultation source and diagnosis explanation on participants’ perceived empathy (*F*_2,240_=8.71; *P*<.001; partial η^2^=0.07) during the medical consultation. Participants did not differ in their perceived empathy for the 3 medical agents when no diagnosis explanation was provided (*P*=.15). However, when the diagnosis was explained, participants perceived significantly more empathy from the human physician (mean 5.08, SD 0.09) than from the AI (mean 4.59, SD 0.09; *P*=.001) and the human-involved AI (mean 4.65, SD 0.09; *P*=.003); however, there was no significant difference between the AI and the human-involved AI (*P*=.99).

Another interaction effect emerged between health-related stigma and diagnosis explanation on the evaluation of source credibility (*F*_1,240_=6.02; *P*=.02; partial η^2^=0.03). When no explanation of the diagnosis was provided, the AIDS group (mean 4.32, SD 0.07) rated the source as significantly more credible than the heart disease group (mean 4.07, SD 0.07; *P*=.01). However, when the diagnosis was explained, no mean difference was found between the 2 types of diseases (*P*=.97).

## Discussion

### Principal Findings

AI is considered a revolutionary technology in the health care sector and is used either independently or in collaboration with humans in medical diagnoses [2]. Nonetheless, the technological expectations of AI from the expert’s perspective do not necessarily translate into an improved evaluation of medical services from the patient’s perspective. This context led to the formulation of the RQs of the study. From the patient’s perspective, can autonomous or human-involved AI replace the role of human physicians at the functional, relational, and emotional levels? How does health-related stigma and its associated need for dehumanization influence patients’ evaluations of medical consultations and preferences for different medical agents? Does providing explanations of the diagnosis equally improve patients’ evaluations of consultations with different medical agents?

The results showed that at the functional level, people were more likely to trust a human physician’s diagnosis than either AI or human-involved AI. However, at the relational and emotional levels, the physician-patient interactions constructed by the 3 medical agents did not differ significantly. Stigma did not significantly affect the evaluation of the medical consultation or the preference for the medical agent, suggesting that in high-stakes medical scenarios, patients’ instrumental need for diagnosis is prioritized over privacy concerns. Furthermore, explaining the diagnostic decision-making process significantly improved patients’ functional, relational, and emotional evaluations of the consultations, implying that AI, human-involved AI, and human physicians should all prioritize providing understandable explanations to patients, which could enhance treatment adherence, strengthen physician-patient relations, and generate positive emotional responses.

### Theoretical Contributions

First, this study contributes to the understanding of HCI and HMC in a medical scenario through the empirical evaluation of 3 influential theories in this field that are divergent in nature, namely, the CASA paradigm, the machine heuristic theory, and the algorithm aversion theory. Previous studies have explored HCI and HMC mostly by analyzing scenarios of web-based content production and moderation, consumer behavior, and so on [5,6]. Which theory best captures the intricacies of the medical scenario remains unclear, especially when considering contextual factors, such as the severe consequences of medical interventions and the attributes of the disease. Moreover, little evidence exists to test, from a user-centered perspective, the perception of human-involved AI, especially vis-à-vis AI and humans; however, this model of human-AI collaboration is increasingly prevalent in medical scenarios. This study found that functionally, people trusted the diagnosis of a human physician more than AI or human-involved AI, and there was no difference between autonomous AI and human-involved AI, supporting the algorithm aversion theory. This may be due to the opacity of AI systems, as evidenced in RQ3 through the observation that explaining the decision-making process significantly enhanced the perceived credibility and persuasiveness of the diagnosis. Another potential explanation is that, at the current stage of AI development, people place greater trust in human expertise than in accurate AI, particularly in decisions that are traditionally made by humans and that carry severe consequences, such as disease diagnosis. Regarding the relational and emotional aspects, no significant difference was found between human-computer (ie, medical consultation with AI or human-involved AI) and human-human (ie, medical consultation with human) interactions. This may be due to a general lack of care on the part of human physicians during medical consultations and AI’s increasing emphasis on creating anthropomorphic relationships.

Second, this study is among the early research endeavors to incorporate the issue of health-related stigma into the evaluation of human-AI interaction [7,8]. It explored whether dehumanized medical consultations were preferred for patients with stigmatized conditions, who might require more anonymity and privacy, or whether human expertise and human touch were preferred in consultations for all diseases. We found that stigma did not significantly influence how users evaluated the consultations or preferred a particular medical agent. This implies that in the highly consequential medical context, the instrumental need for diagnosis outweighs privacy concerns. In the context of illness, patients are keen to receive treatment and less likely to prefer a medical agent simply because of the different levels of privacy exposure that exist between human-AI interaction and human-human interaction. However, including the diagnosis explanation in the analysis (RQ3) revealed an interaction effect between health-related stigma and diagnosis explanation on people’s source credibility ratings. We found that stigma had no significant effect on perceived source credibility when the diagnosis was explained in detail, suggesting that patients are likely to judge source credibility based on the diagnostic explanation when it is provided. However, when patients were only given the name of the disease and treatment options but not an explanation of why this decision was made, more stigma associated with the disease led to higher perceived source credibility from the patient. The aforementioned findings collectively indicate that while overall stigma does not significantly influence individual judgment, when patients are provided solely with the name of the disease and treatment options without an explanation of the diagnostic decision-making process, patients experiencing stigmatized diseases are susceptible to the psychology of shame and the eagerness to find solutions without demanding transparency from the medical agent. This is regardless of whether the agent is an AI, a human-involved AI, or a human physician.

Third, we advanced health communication literature by exploring, from a patient-centered perspective, a potential approach to improving patient-physician communication, which is providing explanations for the diagnostic decision-making process. We found that including a diagnosis explanation significantly improved patients’ evaluations of the consultation at functional, relational, and emotional levels and for all 3 medical agents. This suggests that regardless of the identity of the communicator (AI, human-involved AI, or human), it is not enough for a medical agent to have the expertise (eg, human physicians) or to be highly accurate (eg, AI and human-involved AI). The explanation for the diagnostic decision-making process is critical for patients to improve treatment adherence, strengthen the physician-patient relationship, and build positive emotions throughout the consultation. On the one hand, this sheds light on how to facilitate traditional physician-patient communication, as evidence showed that patients sometimes had no choice but to turn to web-based communities to decipher the medical knowledge and reasoning embedded in the medical disclosure documents [34]. More importantly, it offers insights into the ways to design an AI-powered medical system, which has long been criticized for lacking transparency in making highly consequential decisions. In addition, an interaction effect appeared between diagnosis explanation and consultation source on perceived empathy. When there was no explanation, participants did not differ in their perceived empathy for the 3 medical agents, but when an explanation was provided, participants perceived significantly more empathy from the human physician than from AI or human-involved AI. This suggests that although unraveling the decision-making process is helpful in building effective medical agent–patient communication, it is still easier for human physicians to establish closer and more empathetic interactions with patients than AI-based systems when the same efforts of diagnosis explanation were made.

### Practical Implications

This study contributes to the understanding of the roles of humans and AI in medical diagnosis and identifies key principles for the future application and design of AI medical agents. First, we found that people generally viewed human physicians as more credible and easier to relate to than AI or human-involved AI when the same effort was made (ie, providing the same diagnosis and the same explanation). Even for conditions where we would expect privacy and anonymity to be required (eg, stigma-related diseases), no significant preference for AI-based systems over humans emerged. This suggests that people still prefer human expertise and are more easily moved by human-human interactions in medical consultations. Thus, it is vital to ensure that human physicians assume a pivotal position in medical diagnosis to foster trust and empathy between physicians and patients. As the health care industry progresses toward embracing AI in medical diagnosis, it is advisable to consider incorporating more human elements in the diagnostic process to gain greater user acceptance.

In addition, we propose that the design of AI-based medical agents should be user centered, with the objective of satisfying the user’s need to understand their own conditions and treatment options rather than merely providing conclusions that are accurate but difficult to comprehend. Medical diagnosis has historically prioritized the functional objective of furnishing accurate diagnoses and treatments. Consequently, the design of numerous AI-powered diagnostic applications has centered on enhancing diagnostic precision through processes such as data learning and algorithm iteration. However, it is insufficient for AI to simply be accurate and efficient to be trusted and embraced by patients. From the patient’s or user’s perspective, an inability to comprehend the diagnosis and link their symptoms to the treatment plan may result in reluctance to adhere strictly to the treatment to achieve desired health outcomes. In examining RQ3, we found that opening the black box of the diagnostic decision-making process and providing explanations to help patients understand their health conditions effectively improved their functional, relational, and emotional evaluations of medical consultations. Therefore, designers of AI-powered systems should place greater focus on taking users’ perspectives, opening the black box of decision-making processes, and ensuring that the diagnostic process is understandable to patients. This will not only enhance patient satisfaction and treatment outcomes at the individual level and in health care scenarios, but it also has practical implications for AI-based product design in a broader sense.

### Limitations and Future Research

First, experiment research has its limitations. Because the scenarios are simulated, some participants may react differently in real-life situations. For instance, a distinction may emerge between an individual genuinely at risk of AIDS and a participant simulating such a condition. People who are genuinely at risk may be more likely than normal participants to prefer dehumanization in medical assistance because of their tendency to withhold real private information such as risky sexual behaviors [27]. However, another possibility is that those facing imminent peril may prioritize their survival over privacy concerns, opting to seek help from authoritative human physicians [46], whereas, in role-playing situations, participants are only imagining the stigma but not experiencing actual life threats. Although these 2 opposing situations may counteract the effect of role-playing, future research could use qualitative methods, such as ethnography and interviews, to further elucidate the intricacies of patient psychology and interpret how they may influence the perception of different medical agents.

Second, this research focused on the effects of consultation sources, health-related stigma, and explanation of diagnosis on patients’ evaluations of medical consultations. We believe that these are highly important variables in the evaluation of medical consultations, but other factors, such as disease severity, may also interact with the aforementioned variables and shape patients’ evaluations of medical consultations. The inclusion of more variables, such as disease severity, in the analyses in future studies will provide more in-depth and detailed insights.

Furthermore, while the experimental scenario in this study is medical diagnosis, AI has many other applications in health care, such as health risk analysis and disease screening. Future studies could investigate whether attitudes toward AI vary across different application scenarios.

### Conclusions

With the increasing application of AI in the medical field to provide diagnosis, either independently or in collaboration with humans, this study aims to examine, from the patient’s perspective, whether AI or human-involved AI can replace the role of human physicians in functional, relational, and emotional dimensions. In addition, we aimed to investigate how health-related stigma and explanations of diagnoses affect patients’ evaluation of the medical consultation and their preferences for the medical agent. Findings indicated that individuals exhibited greater trust in diagnoses rendered by a human physician than by AI or human-involved AI, but at the relational and emotional levels, there was no significant difference between the human-AI and human-human interactions. Stigma did not have a significant impact on how people evaluated the medical consultation or contribute to preferring AI-powered systems over humans. However, providing explanations of the diagnosis significantly improved patients’ functional, relational, and emotional evaluations of the consultation for all 3 medical agents. By comparing people’s perceptions of AI, human-involved AI, and human physicians in medical diagnosis, this study contributed to the understanding of human-AI interaction in the medical scenario and empirically tested 3 influential but divergent theories in the field (CASA, machine heuristic theory, and algorithm aversion theory). Furthermore, we advanced health communication studies by incorporating health-related stigma into the evaluation of human-AI interaction, as well as by demonstrating that explaining the diagnostic decision-making process is effective in improving patient-physician communication.

## Appendix

Multimedia Appendix 1 The stimulus materials and the questionnaire used for the experiment.

## Abbreviations

AI: artificial intelligence
CASA: computers are social actors
HCI: human-computer interaction
HMC: human-machine communication
RQ: research question

## Data Availability

The datasets generated during and analyzed during this study are available from the corresponding author on reasonable request.
