# Supplementary material for: Investigating Whether AI Will Replace Human Physicians and Understanding the Interplay of the Source of Consultation, Health-Related Stigma, and Explanations of Diagnoses on Patients’ Evaluations of Medical Consultations: Randomized Factorial Experiment
Source: J Med Internet Res. 2025 Mar 5;27:e66760. doi: 10.2196/66760 (PMC11923482; doi:10.2196/66760)
Supplement: Multimedia Appendix 1 [file jmir_v27i1e66760_app1.docx]

**Examples of stimulus materials.**

**1. An illustration of the medical agent-patient communication interface**

In this study, a common online one-to-one chat interface was utilized. The following example illustrates one of the experimental scenarios, which is a chat between a medical AI and an information seeker concerning symptoms of HIV/AIDS. It should be noted that the chat interface is the same for all the experimental scenarios. Utilizing a question-and-answer format, the patient describes the symptoms they are experiencing to the medical agent and responds to the relevant medical history and other questions posed by the medical agent. The medical agent will then proceed to formulate a diagnosis and suggest treatment options, informed by the symptoms and the patient’s responses.


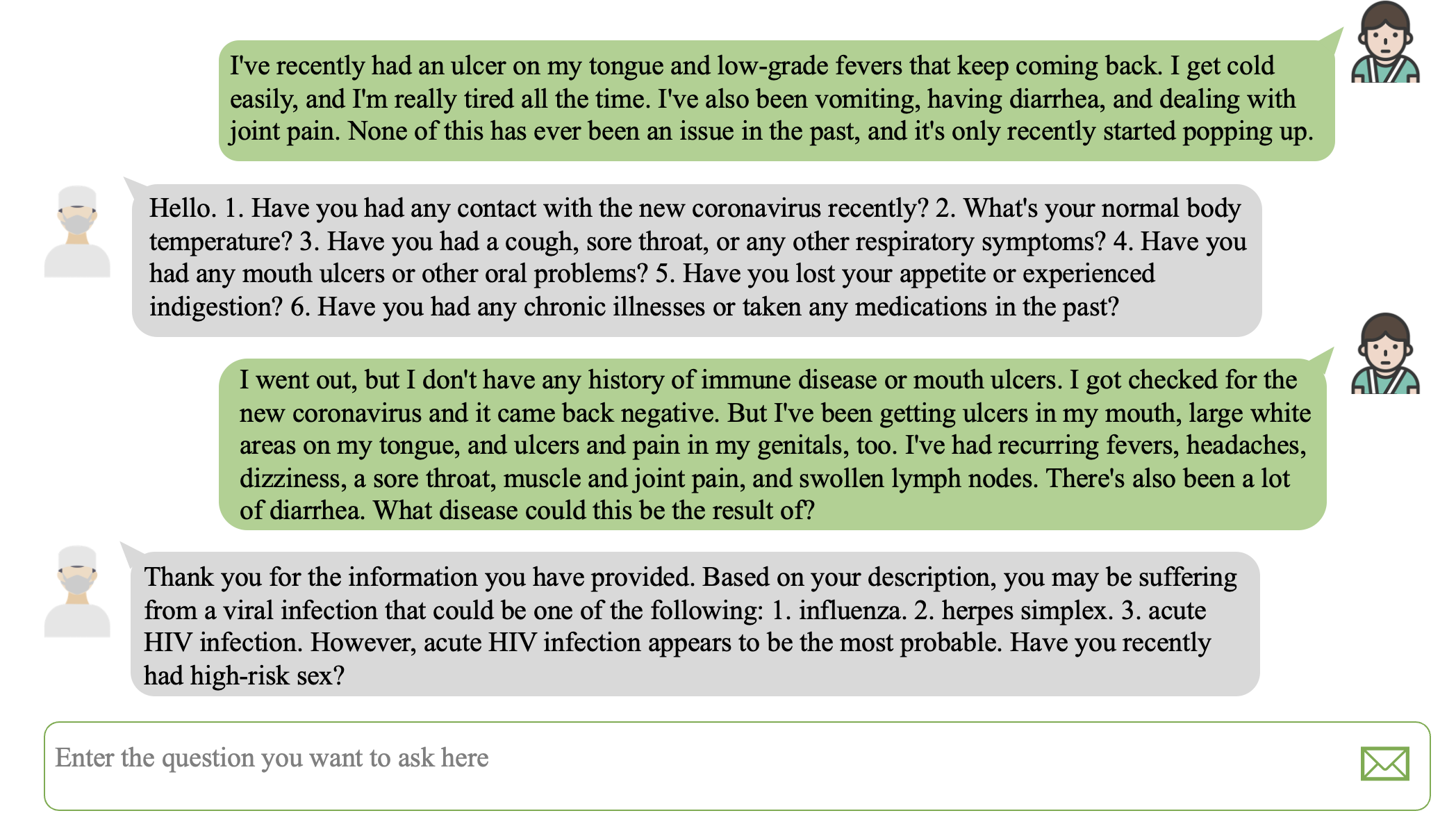


**2. An example of a complete transcript of the conversation between the medical agent and the patient**

Experimental scenario 1.

(1) consultation source: autonomous AI; (2) health-related stigma: high stigma—HIV/AIDS; (3) diagnosis explanation: without explanation.

Scenario introduction (prompt):

You’ve been feeling unwell lately and would like to have a medical consultation to confirm your condition and get advice on treatment. Now, you are having a consultation with *a medical AI*. It learns from past medical data of patients with the same condition, makes a diagnosis based on the symptoms you’ve described, and offers treatment options. *Human physicians are not involved* in diagnosis, medication recommendations or monitoring of the consultation process. Through a series of one-on-one questions and answers, you describe the symptoms you are experiencing to the medical AI, and answer the medical AI’s questions about your relevant medical history and related concerns. The medical AI will then suggest a diagnosis for your condition and treatment options, informed by the symptoms presented and your medical history.

Full script of experimental scenario 1:

**You**: I’ve recently had an ulcer on my tongue and low-grade fevers that keep coming back. I get cold easily, and I’m really tired all the time. I’ve also been vomiting, having diarrhea, and dealing with joint pain. None of this has ever been an issue in the past, and it’s only recently started popping up.

**Autonomous AI**: Hello. 1. Have you had any contact with the new coronavirus recently? 2. What’s your normal body temperature? 3. Have you had a cough, sore throat, or any other respiratory symptoms? 4. Have you had any mouth ulcers or other oral problems? 5. Have you lost your appetite or experienced indigestion? 6. Have you had any chronic illnesses or taken any medications in the past?’

**You**: I went out, but I don’t have any history of immune disease or mouth ulcers. I got checked for the new coronavirus and it came back negative. But I’ve been getting ulcers in my mouth, large white areas on my tongue, and ulcers and pain in my genitals, too. I’ve had recurring fevers, headaches, dizziness, a sore throat, muscle and joint pain, and swollen lymph nodes. There’s also been a lot of diarrhea. What disease could this be the result of?

**Autonomous AI**: Thank you for the information you have provided. Based on your description, you may be suffering from a viral infection that could be one of the following:

1. influenza.

2. herpes simplex.

3. acute HIV infection.

However, acute HIV infection appears to be the most probable. Have you recently had high-risk sex?

**You**: Is there any chance I could get HIV from having unprotected sex? Are my symptoms a sign that I’ve been infected?

**Autonomous AI**: I understand your concern. I strongly recommend that you take the following steps as soon as possible:

1. get tested for HIV

2. seek PEP (Post Exposure Prophylaxis) services.

Stay Safe!

**You**: What are my options if I test positive for HIV?

**Autonomous AI**: You should use an ART regimen and take medications, such as lamivudine, tenofovir, nilavirine, pesatasvir, lopinavir, atazanavir, ladunavir, and darunavir.

**3. Prompts used to introduce different sources of medical consultation**

The autonomous AI scenario: You’ve been feeling unwell lately and would like to have a medical consultation to confirm your condition and get advice on treatment. Now, you are having a consultation with *a medical AI*. It learns from past medical data of patients with the same condition, makes a diagnosis based on the symptoms you’ve described, and offers treatment options. *Human physicians are not involved* in diagnosis, medication recommendations or monitoring of the consultation process. Through a series of one-on-one questions and answers, you describe the symptoms you are experiencing to the medical AI, and answer the medical AI’s questions about your relevant medical history and related concerns. The medical AI will then suggest a diagnosis for your condition and treatment options, informed by the symptoms presented and your medical history.

The human-involved AI scenario: You’ve been feeling unwell lately and would like to have a medical consultation to confirm your condition and get advice on treatment. Now, you are having a consultation with *a human-involved medical AI*. It learns from past medical data of patients with the same condition, makes a diagnosis based on the symptoms you’ve described, and offers treatment options. *Human experts are engaged* in the machine training process of this medical AI, supervise and correct the diagnostic results and treatment recommendations given by the AI. Through a series of one-on-one questions and answers, you describe the symptoms you are experiencing to the human-involved AI, and answer the AI’s questions about your relevant medical history and related concerns. The human-involved AI will then suggest a diagnosis for your condition and treatment options, informed by the symptoms presented and your medical history.

The human physician scenario: You’ve been feeling unwell lately and would like to have a medical consultation to confirm your condition and get advice on treatment. Now, you are having an online consultation with *a physician from the local hospital*. The physician has obtained a Doctor of Medicine degree and possesses considerable expertise in clinical practice. Through a series of one-on-one questions and answers, you describe the symptoms you are experiencing to the physician, and answer the physician’s questions about your relevant medical history and related concerns. The physician will then suggest a diagnosis for your condition and treatment options, informed by the symptoms presented and your medical history.

Note. In the present study, prompts were utilized to introduce the identity of the medical agent (i.e., variable 1, source of medical consultation). The other two variables, i.e., whether the disease was stigmatized and whether an explanation of the diagnosis was provided, were not included in the prompts, but were prompted to the participants throughout the content of the conversation between the medical agent and the patient in the stimulus materials. For a comprehensive overview of the experimental design and execution, please refer to the Methods section of this paper. Furthermore, subsequent to the participants’ engagement with the stimulus materials, manipulation checks were conducted on the three variables. The results indicated that the manipulation was effective in the present study.

**The questionnaire used for the experiment**

**1. Manipulation check**

1.1 The medical agent who provides the diagnosis and treatment advice in the material is:

(1=The medical agent is an autonomous AI. 4=The medical agent is a human-involved AI. 7= The medical agent is a human physician.)

1.2 To what extent is the disease addressed in the material a sensitive issue, socially stigmatized and discriminated against? (1=strongly disagree, 7=strongly agree)

The disease is often associated with shame, social stigma and discrimination.

If I have the disease, I don’t want my friends and neighbors to know about it.

Most people are reluctant to socialize closely with someone who has this condition.

1.3 To what extent did the medical agent in the material provide an explanation of the diagnosis of the disease and the treatment recommendations?

(1= The medical agent did not provide any explanation. 7=The medical agent provided detailed explanation.)

**2. Dependent measures**

2.1 Source credibility

I believe that the medical agent is competent to make a diagnosis and suggest treatment.

I believe that the medical agent can be trusted.

I believe that the medical agent knows a lot.

I believe that the medical agent has goodwill.

2.2 Message credibility

I think the diagnosis and treatment recommendations are accurate.

I think the diagnosis and treatment recommendations are authentic.

I think the diagnosis and treatment recommendations are believable.

2.3 Persuasive effect

I am in agreement with the medical agent’s diagnosis.

I am going to follow the treatment advice.

I will take the medication recommended by the medical agent.

I will seek more information from the medical agent in the future.

I will schedule another appointment with the medical agent if necessary.

2.4 Perceived empathy

The medical agent came across as empathic.

The medical agent understood my feelings.

The medical agent responded appropriately to my feelings and emotions.

The medical agent said the right thing to make me feel better.

The medical agent understood my personal needs.

2.5 Self-disclosure

I feel comfortable disclosing personal information when interacting with the medical agent.

I feel that I can open up to this medical agent about issues that are not easy to discuss with others during the course of the interaction.

I feel that I can talk to this medical agent about my personal life experiences in relation to this illness.

I feel that I can reveal my personal feelings, concerns and thoughts to the medical agent.

2.6 Psychological distance

Please choose the picture that best described the relationship between you and the medical agent. (1=farthest, 7=closest)

2.7 Emotional responses

Do the following statements describe your feelings after the conversation with the medical agent?

I feel more optimistic after the consultation.

I feel more guilty after the consultation.

**3. Demographics**

Sex (Male, Female)

Age
